# Qing-Chang-Hua-Shi granule ameliorates experimental colitis by modulating *Lactobacillus gasseri*-mediated ferroptosis metabolic pathway

**DOI:** 10.1186/s13020-025-01317-5

**Published:** 2026-01-26

**Authors:** Cheng Cheng, Lei Zhu, Jingyi Hu, Wan Feng, Weiyang Li, Ryan Au, Yanan Li, Feng Xu, Yuguang Wu, Yuan Cui, Zhe Di, Bin Li, Yongchang Miao, Yao Lin, Lilin Ge, Hong Shen

**Affiliations:** 1 Jiangsu Province Engineering Research Center of TCM Health Preservation, Nanjing University of Chinese Medicine, Nanjing, 210023 China; 2 Affiliated Hospital of Nanjing University of Chinese Medicine, Nanjing, 210023 China; 3 Academy of Chinese Culture and Health Sciences, Oakland, CA USA; 4 College of Integrative Medicine, Second Affiliated Hospital of Fujian University of Traditional Chinese Medicine, Fujian-Macao Science and Technology Cooperation Base of Traditional Chinese Medicine-Oriented Chronic Disease Prevention and Treatment, Fujian University of Traditional Chinese Medicine, Fuzhou, Fujian China; 5 Fujian-Hong Kong-Macau-Taiwan Collaborative Laboratory for the Inheritance and Innovation of Traditional Chinese Medicine, Fujian University of Traditional Chinese Medicine, Fuzhou, Fujian China; 6 Department of General Surgery, Affiliated Lianyungang Clinical College of Nantong University, Lianyungang, 222006 China

**Keywords:** Qing-Chang-Hua-Shi granule, Colitis, *Lactobacillus gasseri*, Ferroptosis

## Abstract

**Background:**

Ulcerative colitis (UC) is a chronic inflammatory disorder marked by epithelial barrier disruption and persistent intestinal inflammation. Despite extensive research, its complex etiology continues to pose therapeutic challenges. Ferroptosis, an iron-dependent form of regulated cell death driven by lipid peroxidation, has recently been implicated in UC pathogenesis. Additionally, the gut microbiota and its metabolites play a pivotal role in maintaining intestinal homeostasis and barrier integrity.

**Purpose:**

This study aimed to investigate the therapeutic potential of a phytotherapeutic agent QCHS to alleviate UC by modulating ferroptosis and the microbiota-metabolome axis, with a particular focus on the role of *Lactobacillus gasseri* (*L. gasseri*).

**Methods:**

A DSS-induced UC mouse model was used to evaluate QCHS efficacy. Gut microbial composition and metabolomic alterations were analyzed via 16S rDNA sequencing and UHPLC-MS/MS. *L. gasseri* was cultured in vitro to assess the impact of QCHS on its growth. RSL3-induced cell death was modeled in NCM-460 cells and ferroptosis-related changes were examined using transmission electron microscopy, immunohistochemistry, quantitative PCR, and Western blotting.

**Results:**

QCHS significantly mitigated DSS-induced ferroptosis in colonic tissues, with *L. gasseri* identified as a key mediator. Notably, *L. gasseri* was found to act as a novel ferroptosis inhibitor. In vitro studies confirmed that *L. gasseri* suppressed RSL3-induced ferroptosis in NCM-460 cells via activation of the GSH/GPX4 pathway.

**Conclusion:**

This study provides compelling evidence for the regulatory role of QCHS on the microbiota-metabolome axis and ferroptosis in UC. It also uncovers a novel function of *L. gasseri* as a ferroptosis inhibitor, offering promising insights into microbiota-targeted and ferroptosis-modulating therapeutic strategies for UC.

**Graphical Abstract:**

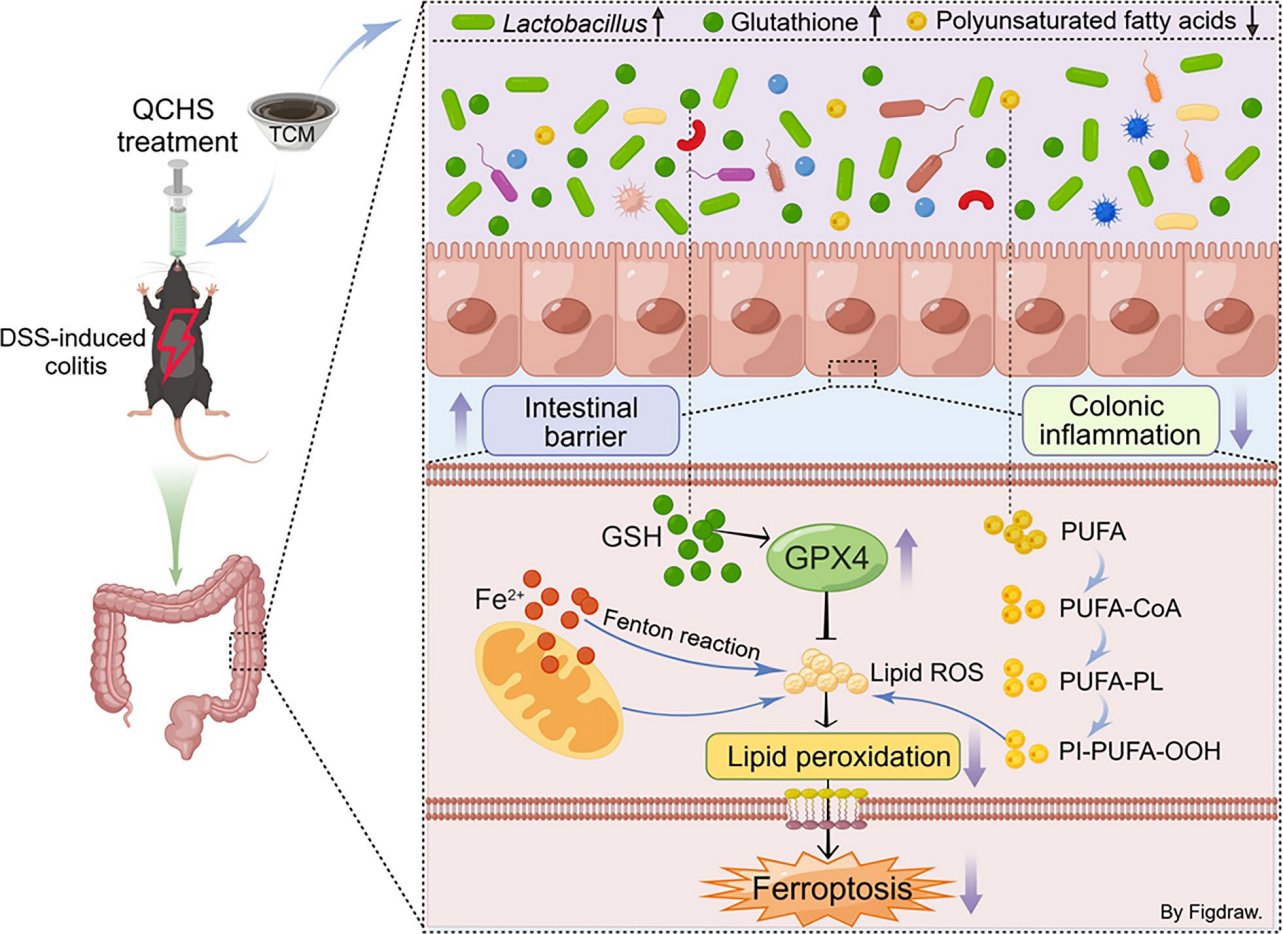

## Introduction

Ulcerative colitis (UC), characterized by chronic intestinal inflammation and epithelial barrier dysfunction, remains a therapeutic challenge due to its complex etiology involving dysregulated immune responses, microbiota dysbiosis, and aberrant cell death pathways [1, 2]. Among emerging mechanisms, ferroptosis—an iron-dependent form of regulated cell death driven by lipid peroxidation—has recently been implicated in UC pathogenesis [3, 4]. The gut microbiota plays a crucial role in maintaining intestinal homeostasis, and disruptions in its composition and its metabolism can result in pathological damage to the intestines [5, 6]. Metabolites act as important mediators of host-microbe interactions and are essential for the maintenance of the gut barrier [7, 8]. Targeting the microbiota-metabolic axis has emerged as a promising approach for managing UC. For instance, bile acids derived from the gut microbiota can increase the proportion of regulatory T cells in the colon of mice, thereby reducing their susceptibility to colitis [9]. Short-chain fatty acids (SCFAs) produced by the microbiota can promote intestinal barrier repair through a variety of mechanisms, including activating inflammasomes and affecting the secretion of interleukin (IL)-18, promoting epithelial cell migration, regulating tight junction protein expression, activating G protein-coupled receptors, and inhibiting tissue protein deacetylases [10, 11]. Additionally, studies have shown that fatty acids produced by gut microbes, especially unsaturated fatty acids, are closely associated with the inflammatory state of IBD [12, 13].

Ferroptosis, a recently discovered form of regulated cell death (RCD), is characterized by iron dependence, lipid peroxidation, increased reactive oxygen species (ROS), and disruption of mitochondrial metabolism [14, 15]. Glutathione peroxidase 4 (GPX4) is a key antioxidant enzyme that utilizes glutathione (GSH) as a substrate to catalyze the reduction of lipid peroxides to alcohol. The production of GSH relies on the cystine/glutamate antiporter system (System Xc−). Inhibition of GPX4/GSH or System Xc− activity is considered a central mechanism in the induction of ferroptosis [16, 17]. Ferroptosis has been implicated as a potential pathogenic factor in IBD [18]. Studies have demonstrated ferroptosis-induced damage in both UC patients and colitic mice, and ferroptosis inhibitors are being explored as potential therapeutic agents for UC [19–21]. However, the relationship between the microbiota and ferroptosis in UC treatment remains largely unexplored.

A phytotherapeutic agent, Qing-Chang-Hua-Shi granule (QCHS) is a traditional Chinese medicine (TCM) formula widely used in China for the treatment of UC. It is composed of Huanglian (*Coptis chinensis* Franch.), Huangqin (*Scutellaria baicalensis* Georgi), Baijiangcao (*Patrinia scabiosaefolia* Fisch.), Danggui (*Angelica sinensis* (Oliv.) Diels), Baishao (*Paeonia lactiflora* Pall.), Diyu (*Sanguisorba officinalis* L.), Zicao (*Lithospermum erythrorhizon* Siebold & Zucc.), Qiancao (*Rubia cordifolia* L.), Baizhi (*Angelica dahurica* (Fisch, ex Hoffm.) Benth.et Hook.f.), Muxiang (*Aucklandia lappa* Decne.), and Gancao (*Glycyrrhiza uralensis* Fisch.) in a ratio of 6:10:15:10:20:10:10:20:12:6:6. Currently, there are limited randomized controlled and double-blind clinical trials of traditional Chinese medicine for ulcerative colitis, while QCHS has been proven to have a clear effect on clinical remission and mucosal healing in patients with moderately active UC [22, 23]. Our previous studies have shown that QCHS can regulate T cell differentiation and has superior anti-inflammatory effects compared to 5-aminosalicylic acid and sulfasalazine in experimental colitis models [24, 25]. However, the effect of QCHS on the microbiota and metabolism requires further investigation.

In this study, we evaluated the effects of QCHS on the gut microbiota and metabolism in UC mice. We demonstrated that QCHS acts as an intestinal microecological regulator by increasing the abundance of *Lactobacillus gasseri* (*L. gasseri*) and altering the profile of ferroptosis-related metabolites in feces. We also explored the correlation between *L. gasseri* and ferroptosis, and for the first time, we report that *L. gasseri* can ameliorate DSS-induced colitis by inhibiting ferroptosis. Our findings reveal a novel mechanism of action for QCHS and *L. gasseri*, suggesting that ferroptosis-targeted herbal medicine or probiotics could serve as a new treatment strategy for UC.

Here, we hypothesize that QCHS ameliorates UC by reshaping the gut microbiome to inhibit ferroptosis. By integrating 16S rDNA sequencing, metabolomics, and functional assays, we reveal that QCHS enriches *Lactobacillus gasseri*, which drives GSH-dependent ferroptosis suppression. Our findings bridge the gap between TCM and modern mechanistic research, highlighting microbiota-ferroptosis axis as a novel target for UC therapy.

## Materials and methods

### Reagents

DSS (36,000–50,000 Da, CAS: 216011080) was obtained from MP Biomedicals. LPS (L2630) and FITC-Dextran (60842-46-8) were purchased from Sigma-Aldrich. cDNA Synthesis Kit (R211-01), qRT-PCR SYBR Green Kit (Q221-01), and CCK-8 Cell Counting Kit (A311-01) were purchased from Vazyme. Fetal Bovine Serum (04-001-1ACS), Trypsin EDTA solution (03-050-1A), and RPMI Medium 1640 (01-100-1ACS) were purchased from Biological Industries. Antibodies against FTH1 (#4393) and β-Actin (#3700) were provided by CST. Antibodies against ZO-1 (sc-33725) was provided by Santa Cruz Biotechnology. Antibodies against Claudin-1 (ab211737), Claudin-5 (ab131259), and Muc-2 (ab272692) were purchased from Abcam. Antibodies against 4-HNE (bs-6313R) were purchased from Bioss. Antibodies against ACSL4 (66617-1), GPX4 (22401-1), and the secondary antibodies were purchased from Proteintech. H&E (G1003), Alcian blue (G1027), and Prussian blue staining kit (G1029) were provided by Servicebio. SP Rabbit & Mouse HRP Kit (DAB, CW2069S) was purchased from CWBio. Iron Assay kit (TC1015) was provided by LEAGENE. Total Glutathione Assay Kit (S0052) and Lipid Peroxidation MDA Assay Kit (S0131S) were provided by Beyotime. All primers were provided by Generay Biotech (Shanghai).

### Preparation of Qing-Chang-Hua-Shi granule

The 11 components of QCHS were provided by Jiangyin Tianjiang Pharmaceutical St (Jiangsu, China) in the form of granules. The “gram weight” refers to the equivalent crude herbal material. Granules were dissolved in double‑distilled water (ddH_2_O) to prepare a stock solution, and then diluted to three working concentrations (0.95, 1.9, and 3.8 g/mL). The dosing regimen was determined based on human equivalent dose (HED) calculations using body surface area conversion from the clinical dose (125 g per 60‑kg adult). Accordingly, the medium dose in mice corresponds to the human dose, while the low and high doses were set proportionally (9.5, 19, and 38 g/kg/day).

### Animal experiments

All mice (C57BL/6 J, male, 20–22 g) used were purchased from Zhejiang Vital River Laboratory Animal Technology Co., Ltd and were raised under SPF conditions at Nanjing University of Chinese Medicine (A standard 12 h light/dark cycle, license number: SYXK (苏) 2018–0049). The experiments were approved by the Animal Ethics Committee of NJUCM (Application Number: 202203A069) on 8th Mar, 2022.

For experiments involving QCHS treatment, mice were randomly divided into 5 groups (n = 8): control group (Ctrl), model group (DSS), low-dose QCHS group (QCHS-L), medium-dose QCHS group (QCHS-M), and high-dose QCHS group (QCHS-H). UC model was established using 3% DSS (dissolved in drinking water) for 7 days. Mice were then given different doses of QCHS (9.5, 19, 38 g/kg/day), or double distilled water (ddH_2_O) for another 7 days by gavage. For experiments involving *L. gasseri* intervention, mice were given 3% DSS with or without *L. gasseri* (5 × 10^8^ CFU) for 7 days. The disease activity index (DAI) was determined using the body weight, fecal properties, and blood in the stool, as described in a previous publication [26]. The serum, colon tissue, and feces were collected for further analysis.

### qRT-PCR

Total RNA was extracted and subsequently reverse-transcribed into complementary DNA (cDNA) using a commercial cDNA Synthesis Kit. Quantitative real-time PCR (qRT-PCR) was performed on a LightCycler^®^ 96 system (Roche) with SYBR Green Master Mix (Vazyme) to determine the cycle threshold (Ct) values. β-actin or GAPDH served as internal reference genes for normalization. All primers were designed and synthesized by Generay Biotech (Shanghai, China).

### Measurement of FITC-Dextran 4 leakage

Mice were fasted for 6 h before being gavaged with FITC-Dextran (40 mg/mL). After 4 h, serum was collected and the fluorescence value of serum samples were measured at 500 nm. A standard curve was drawn with FITC standards and the sample concentrations were calculated based on the fluorescence values. All samples were protected from light during the experiment.

### Histological evaluation of colitis

Colon tissue segments (0.5 cm) were washed with PBS and fixed in paraformaldehyde for 48 h before being embedded in paraffin and cut into 4 μm thick sections for further analysis. H&E, Alcian blue, and Prussian blue staining were performed according to the staining kits’ protocol (Servicebio) after deparaffinization. Images were captured with a light microscope (Leica). The histological scores were performed as previously described based on crypt loss, lymphoid follicle formation, and inflammatory infiltration [26].

Histological scores were determined in a blinded manner by two independent investigators based on the sum of the epithelium and infiltration scores. Epithelium score: 0 = normal; 1 = loss of goblet cells; 2 = loss of goblet cells in large areas; 3 = loss of crypts; 4 = loss of crypts in large areas. Infiltration score: 0 = normal; 1 = infiltration around crypt bases; 2 = infiltration reaching the muscularis mucosae; 3 = extensive infiltration reaching the muscularis mucosae; 4 = infiltration of the submucosa.

### Immunostaining analysis

After deparaffinization, antigen retrieval of colon tissue sections was performed using sodium citrate solution. Next, the sections were blocked with 2% BSA at 37 °C for 1 h and then incubated with anti-Muc-2 (1:200), anti-Claudin-1 (1:200), and anti-4-HNE (1:100) at 4 °C overnight. After washing with PBS for 3 times, the tissue sections were incubated with the corresponding secondary antibodies at 37 °C for 1 h. For 4-HNE staining, sections were visualized according to the standard methods of the diaminobenzidine (DAB) solution kit. For Muc-2 and Claudin-1 staining, an anti-fade reagent was added after counterstaining the nucleus with DAPI. All images were analyzed using an inverted fluorescence microscope (Leica, DMi8).

### Transmission electron microscopy (TEM)

Colon tissues were segmented into 1 mm^3^ sections on ice and fixed with 2.5% glutaraldehyde at 4 °C overnight. Samples were then dehydrated in a gradient of ethanol concentrations and acetone, and then embedded in resin. 60–80 nm ultra-thin sections were cut from the resin block and transferred to 150 mesh copper grids coated with formvar film. The samples were stained with 2% uranyl acetate saturated alcohol solution followed by 2.6% lead citrate solution. Finally, the samples were observed using a transmission electron microscope (HITACHI HT7800, 120 kV).

### 16S rDNA sequence analysis

The microbial sequencing analysis was performed by Allwegene Tech. Briefly, the genomic DNA was extracted and the integrity of DNA was inspected using 1% agarose gel electrophoresis. The V3-V4 regions of 16S rDNA gene were amplified and the PCR products were recovered using 1% agarose gel electrophoresis. Next, DNA were purified with Agencourt AMPure XP Nucleic Acid Purification Kit. DNA sequencing was performed based on the Illumina MiSeq platform (PE300). Chimeras and short sequences were removed from sequencing data to obtain high-quality sequences and operational taxonomic units (OTUs) were generated. Finally, OTUs with a similarity level of less than 97% were used for further bioinformatics analysis.

### Quantification of *Lactobacillus gasseri* in stool

The genomic DNA of feces was extracted using TIANamp Genomic DNA Kit (DP304, TIANGEN). PCR amplification of DNA was performed using *L. gasseri-*specific primers (F: 5’ -AATACTCCCGAAGCACGTCA-3’, R: 5’-TCATTGTGTTTGGCAATCGT-3’). PCR products were then checked with 1% agarose gel electrophoresis and recovered using the Magbead Gel Extraction Kit (CWBIO). Purified DNA was cloned using DH5α (CWBIO), and the plasmid was extracted as a standard for PCR quantification. The plasmid standard was diluted tenfold from 10^1^–10^5^, and 2μL of gradient was used as a template to establish a standard curve. The samples and standard DNA underwent Realtime PCR reaction, and then the DNA quantity was calculated according to the standard curve. All experiments were repeated three times.

### Bacterial strain and culture conditions

The *L. gasseri* (BNCC135322) strain was purchased from Bena Culture Collection (Henan, China) and confirmed by 16S rDNA sequencing in Allwegene Tech, Ltd. (Beijing, China). *L. gasseri* were grown anaerobically in MRS broth at 37°C. The bacteria were centrifuged (4000 × g, 10 min) and washed twice with sterile phosphate-buffered saline (PBS) solution, and finally resuspended in sterile PBS before use.

### Detection of *L. gasseri* growth curve

The fresh *L. gasseri* suspension was inoculated into 200 mL of MRS liquid medium at a 2% (v/v) inoculation volume. The OD600 value was measured every 3 h (repeated 3 times with the average value taken), and the 24-h growth curve of *L. gasseri* was plotted according to the incubation time and absorbance values.

To determine QCHS’s effects on the growth of *L. gasseri*, the bacteria were cultured in MRS liquid medium with or without different concentrations of QCHS (0, 5, 10, 50, 100, 250, 500, 1000, 2500, 5000 μg/mL) for 24 h from the start of inoculation. The OD600 value of the culture medium was measured and the survival rate of bacteria was calculated using the normal MRS culture group as a reference.

### Non-targeted metabolomic analysis

Fecal samples (200 mg) were mixed with pre-chilled 80% methanol, vortexed, incubated on ice for 5 min, then centrifuged at 15,000 × g at 4 °C for 20 min. The supernatant was collected and then diluted with mass spectrometry-grade water so that the final concentration of methanol was 53%. Samples were then centrifuged at 15,000 × g at 4 °C for 20 min. The supernatant was collected and injected into the UHPLC-MS/MS system (Thermo Fisher) with a Hypesil Gold column (C18, 100 × 2.1 mm, and 1.9 μm) for analysis. The mass spectrometer was operated in both positive and negative ionization mode with a mass range of 100 to 1500. The LC–MS/MS data were processed using Compound Discoverer 3.1 (CD3.1, Thermo Fisher) to perform peak alignment, peak picking, and quantification for each metabolite. The peaks were matched with mzCloud (https://www.mzcloud.org/),mzVault, and MassList databases to obtain accurate qualitative and relative quantitative results. The metabolites were annotated using the KEGG database and statistical analysis was performed using the statistical software R (R version R-3.4.3), Python (Python 2.7.6 version), and CentOS (CentOS release 6.6). Metabolomics analysis was completed by Novogene Co., Ltd. (Beijing, China).

### Assessment for GSH, MDA, and iron levels

Protein samples were collected from colon tissues or NCM-460 cells and the concentrations were determined using the BCA Protein Assay Kit (Beyotime). The levels of Glutathione (GSH) were tested using a Total Glutathione Assay Kit (S0052, Beyotime). The contents of MDA were detected through the Lipid Peroxidation MDA Assay Kit (S0131S, Beyotime). An Iron Assay kit (TC1015, LEAGENE) was used to determine the iron levels of colon or NCM-460 cells. The final unit concentrations were calculated based on the sample protein concentrations.

### Cell culture and model establishment

Normal human colonic epithelial NCM-460 cells were cultured with RPMI 1640 medium (10% fetal bovine serum) under standard conditions. Inflammation was induced in NCM-460 cells using 1 μg/mL LPS. Briefly, cells (2 × 10^5^) were treated with *L. gasseri* (10^5^–10^7^ CFU/mL) for 12 h before LPS stimulation. RSL3 (3 μM) was utilized to induce ferroptosis in cells after *L. gasseri* treatment and the cells without any treatment were used as a negative control. Cell viability was detected using the standard procedures of the CCK8 assay (Vazyme). Total protein and RNA were collected for further analysis.

### Cell viability assay

NCM-460 cells (1 × 10^4^) were plated into 96-well plates and incubated with *L. gasseri* (10^2^–10^7^ CFU/mL) for 24 h. Next, 10 μL CCK-8 was added into the plate and the absorbance of cells was measured at 450 nm after 2 h of incubation.

### Statistical analysis

All results were displayed as mean ± SEM. Statistical difference between multiple groups were analyzed by one-way ANOVA and *P* < 0.05 was considered significant. All statistical data were analyzed with GraphPad Prism 9.0.

## Results

### QCHS alleviated DSS-induced colitis in mice

**Fig. 1:**
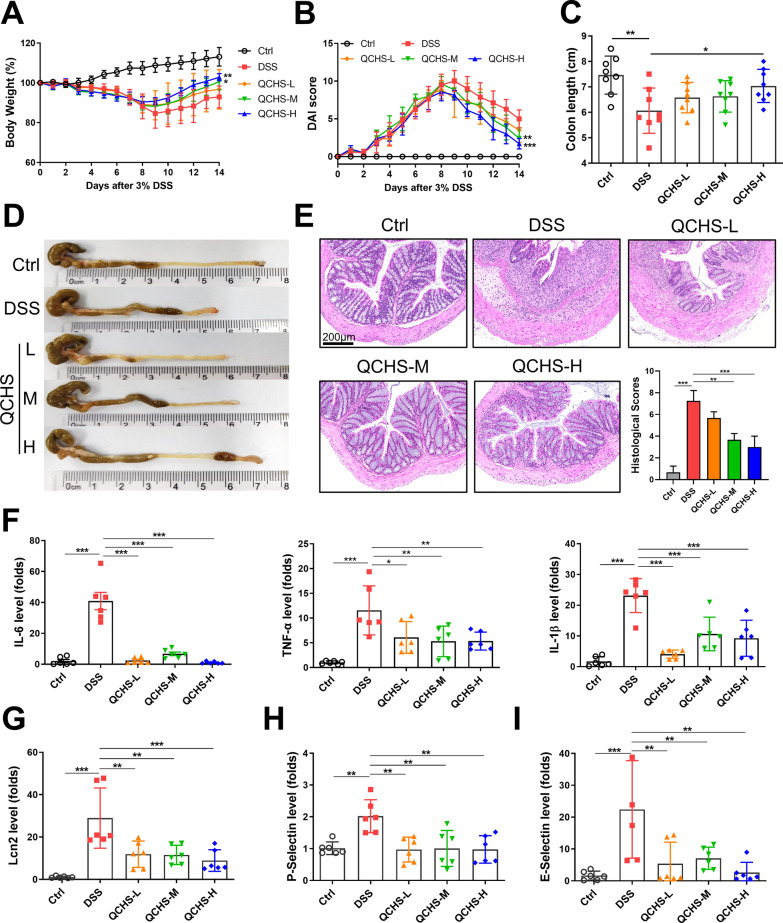
QCHS alleviated DSS-induced colitis in mice. **A** Body weight change and **B** Disease activity index (DAI) evaluation. **C**–**D** Colon length. **E** H&E staining and pathological scoring of colon tissue, Scale bar: 200 μm. **F** The mRNA levels of IL-6, TNF-α, and IL-1β were analyzed by real-time PCR. **G**–**I** The levels of inflammatory marker Lipocalin 2 (Lcn2) and cell adhesion molecules (P-selectin, E-selectin) were also detected by real-time PCR. All data are shown as mean ± SEM. *P < 0.05, **P < 0.01, ***P < 0.001 (one-way ANOVA)

We first evaluated the therapeutic effect of QCHS using a 3% DSS-induced UC model. Mice in the QCHS-treated groups exhibited attenuated weight loss, lower DAI scores, and increased colon length compared to the DSS-only group (Fig. 1A–D). The protective effects of QCHS were also shown to be dose-dependent. Histopathological images revealed that colonic mucosal disruption, inflammatory cell infiltration, and goblet cell loss were all restored in QCHS-treated mice. Moreover, the pathological scores of the QCHS-treated group were significantly lower than those of the DSS group (Fig. 1E). As shown in Fig. 1F–G, the levels of pro-inflammatory cytokines (TNF-α, IL-1β, and IL-6) and the inflammatory marker Lipocalin 2 (Lcn-2) were significantly increased in the colon of UC mice, and QCHS treatment reversed these abnormal inflammatory responses. QCHS treatment also reversed the DSS-induced abnormal secretion of P-Selectin and E-Selectin, adhesion molecules involved in the activation of cell proliferation, differentiation, and inflammation (Fig. 1H–I). These results indicate that QCHS treatment significantly alleviated the DSS-induced colonic inflammatory response in mice.

### QCHS improved intestinal barrier function in UC mice

**Fig. 2:**
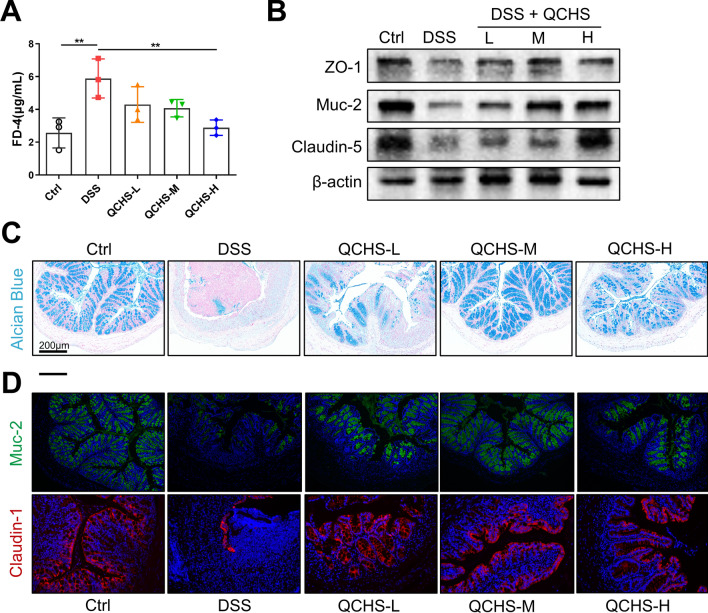
QCHS improved the integrity of the intestinal barrier in UC mice. **A** Intestinal permeability was measured by FITC-Dextran 4 (FD-4) concentration in the serum. **B** The protein levels of ZO-1, Muc-2, and Claudin-5 were examined by western blotting. **C** Alcian blue staining of colon tissue, scale bar: 200 μm. **D** Immunofluorescence images of Muc-2 and Claudin-1 in colon sections are shown. All data are shown as mean ± SEM. *P < 0.05, **P < 0.01, ***P < 0.001(one-way ANOVA)

Disruption of intestinal barrier function is a key characteristic of UC. Therefore, we assessed the protective effect of QCHS on the intestinal barrier in UC mice. The concentration of FD-4 in the serum of QCHS-treated mice was significantly lower compared to the DSS group (Fig. 2A), suggesting that QCHS reduced intestinal permeability in UC mice. The protein levels of Mucin-2 (Muc-2) and tight junction proteins (ZO-1, claudin-5) in the colon of UC mice were significantly increased by QCHS treatment (Fig. 2B). Alcian blue staining revealed a significant increase in colonic mucin production after QCHS treatment compared to the DSS group (Fig. 2C). Furthermore, immunofluorescence staining showed that the expression of Muc-2 and Claudin-1 was reduced in colitic mice, but QCHS significantly restored their expression (Fig. 2D). These results suggest that QCHS can improve intestinal barrier function in UC mice.

### QCHS reprogrammed the gut microbiota and increased the relative abundance of *L. gasseri*

**Fig. 3:**
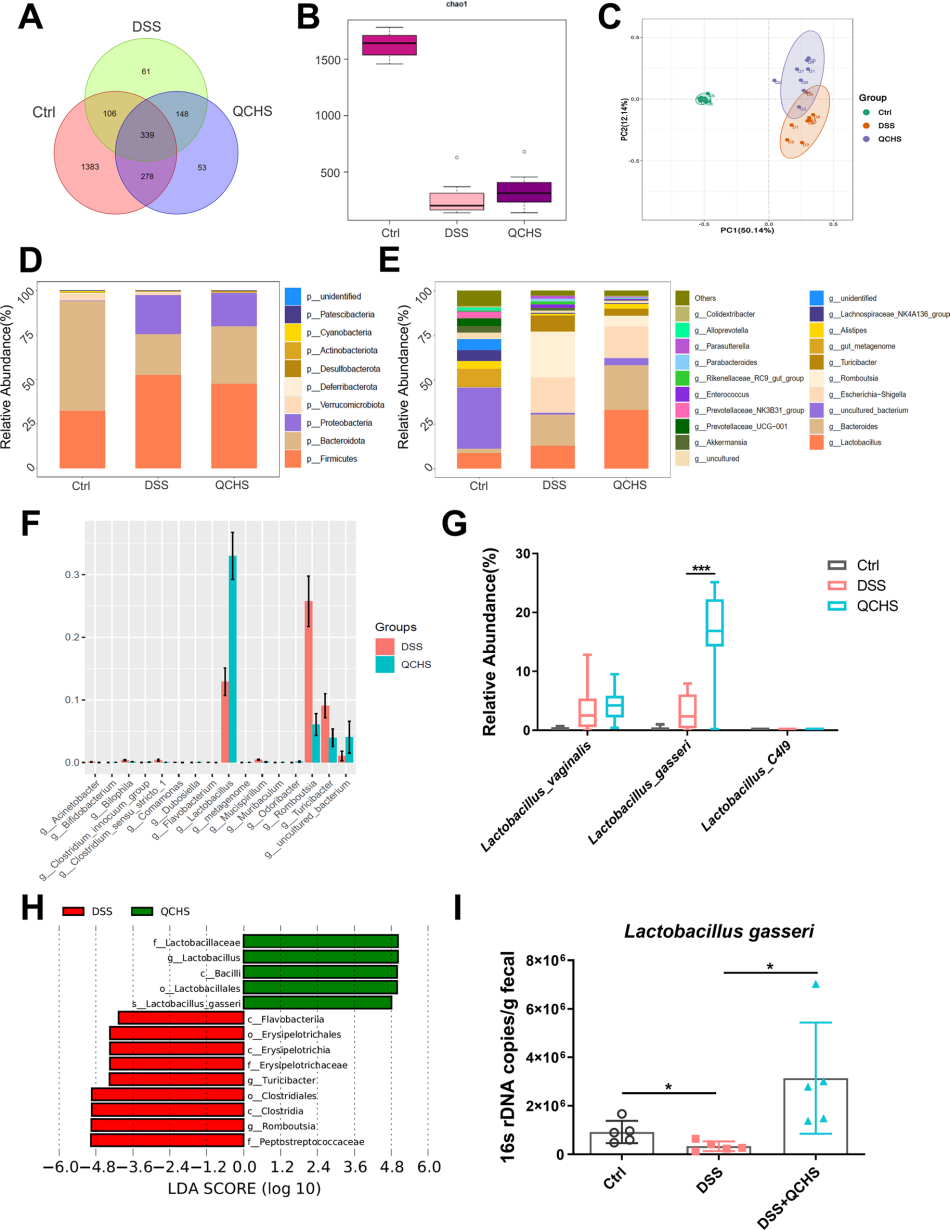
QCHS reprogrammed the gut microbiota and increased the relative abundance of *L. gasseri*. **A** The overlap of OTUs across groups were presented using a Venn diagram (n = 8). **B** Alpha diversity indices (Chao1) and **C** Beta diversity index (principal co-ordinates analysis, PCoA) of gut microbial communities. **D** The relative bacterial abundance at the phylum level and **E** genus level were shown. **F** Wilcoxon test analyzed differential bacteria at the genus level of two groups. **G** The relative abundance of *Lactobacillus* at the species level. **H** LEfSe analysis identified the enriched bacteria in the gut microbiome of the two groups. (LDA score > 4 and a significance of *P* < 0.05 determined by the Wilcoxon test.) **I** 16 s rDNA copies of *L. gasseri* in feces were measured by quantitative PCR. All data are shown as mean ± SEM. *P < 0.05, **P < 0.01, ***P < 0.001(one-way ANOVA)

The gut microbiome plays a crucial role in maintaining intestinal homeostasis. Therefore, we explored the effect of QCHS on the gut microbial composition in UC mice using 16S rDNA sequencing. The Venn diagram shown in Fig. 3A illustrates the differences in the intestinal microbiota among the three groups of mice. The Chao1 index revealed that the alpha-diversity of the microbiome was affected by QCHS in mice with DSS-induced colitis (Fig. 3B). Principal coordinates analysis (PCoA) showed differences in the gut microbial structure between the control group, the DSS group, and the QCHS group, indicating that QCHS treatment significantly altered the gut microbiota structure in UC mice (Fig. 3C).

In Fig. 3D, the bar plot shows that *Bacteroidota*, *Firmicutes*, and *Proteobacteria* were the dominant phyla, and the ratio of *Bacteroidota* to *Firmicutes* was reversed after QCHS treatment. At the genus level (Fig. 3E), the bacteria with the highest relative abundance were *Lactobacillus*, *Bacteroides*, *Escherichia-Shigella*, *Romboutsia*, and *Turicibacter*. The Wilcoxon test was used to compare the microbiota between the QCHS and DSS groups, showing significant differences. The results indicated that QCHS significantly decreased the relative abundance of *Romboutsia* and *Turicibacter* and dramatically increased the relative abundance of *Lactobacillus* (Fig. 3F). More importantly, species-level analysis revealed that the relative abundance of *Lactobacillus gasseri* was significantly elevated by QCHS treatment (Fig. 3G). LEfSe analysis (LDA score > 4) was performed to further explore which bacteria were significantly affected by QCHS. Notably, there were significant differences in the relative abundance of *Lactobacillus* at different taxonomic levels after QCHS treatment (Fig. 3H). Quantitative PCR detection further confirmed that *L. gasseri* was significantly enriched by QCHS (Fig. 3I). These results collectively indicate that QCHS altered the gut microbial composition and significantly increased the relative abundance of *L. gasseri* in colitic mice.

### QCHS altered ferroptosis metabolism and increased Glutathione levels

**Fig. 4:**
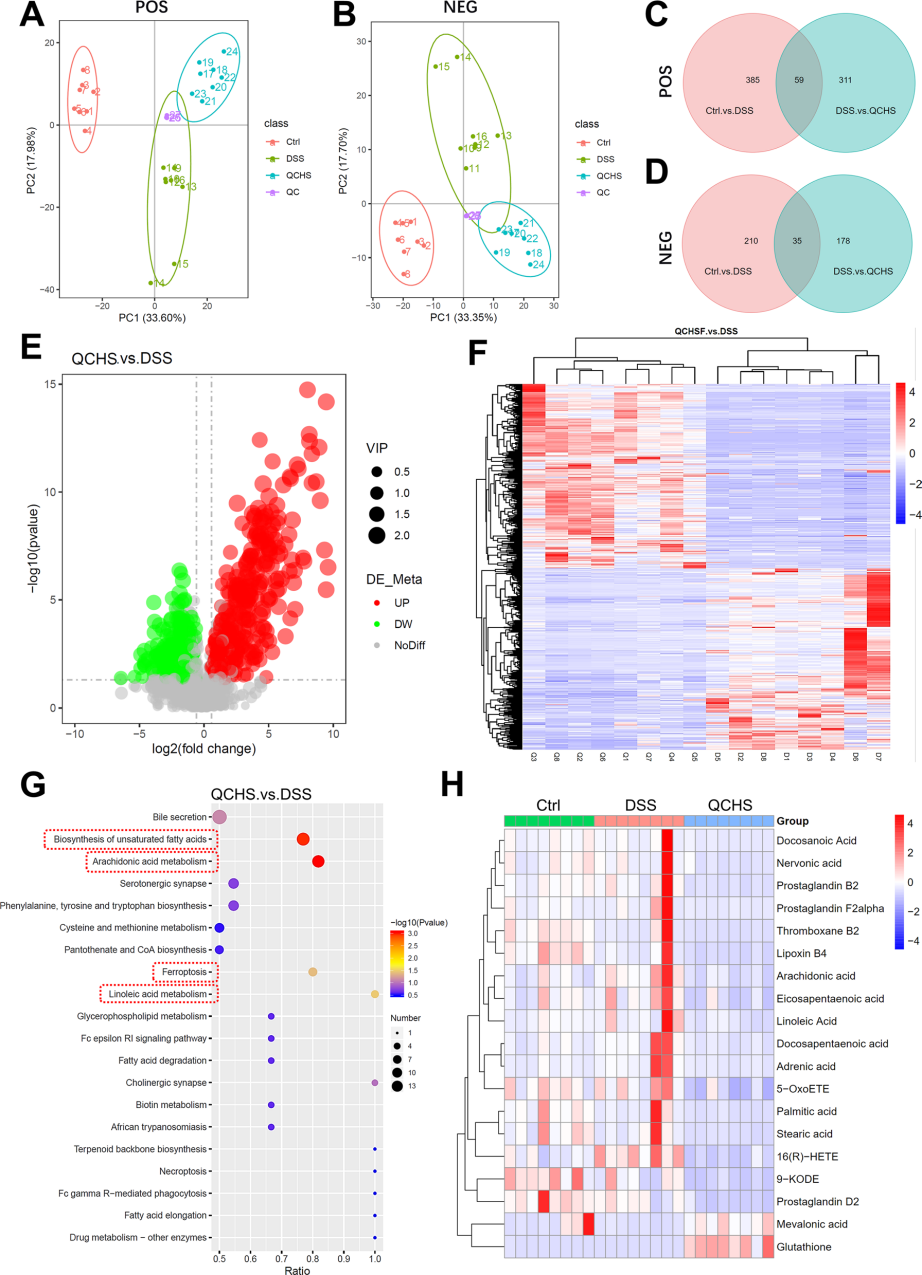
Ferroptosis-related metabolic pathways were significantly enriched after QCHS treatment. **A**–**B** PCA analysis of the metabolites in feces under positive and negative ion mode demonstrated distinct metabolite composition between different groups (n = 8). **C**–**D** Venn diagram analysis of differential metabolites in two ion modes. **E** Volcano plot showing metabolites with significant changes between QCHS and DSS groups, with each dot representing a metabolite. **F** Clustering heat map of total differential metabolites in the feces of three groups. **G** KEGG Pathway enrichment analysis of differential metabolites (top 20, metabolic pathways with significant differences are marked with red boxes). **H** Cluster heatmap of the metabolites in four metabolic pathways with significant differences

Metabolites are important mediators through which the gut microbiota exerts its effects. Therefore, we used UHPLC-MS/MS to examine the metabolomic changes in QCHS-treated UC mice. PCA analysis in both positive and negative ion modes demonstrated that the metabolic profiles of the fecal matter differed among the experimental groups (Fig. 4A-B). The Venn diagram under the positive ion mode showed that 311 metabolites were significantly altered in the feces of mice after QCHS treatment (Fig. 4C), while the negative ion mode revealed 178 differential metabolites between the QCHS and DSS groups (Fig. 4D). The volcano plot shows the overall differences in fecal metabolites between the QCHS and DSS groups when both positive and negative ions were combined (Fig. 4E). Cluster analysis was used to examine the expression levels of all differential metabolites, as shown in Fig. 4F.

The Kyoto Encyclopedia of Genes and Genomes (KEGG) database is a powerful tool for analyzing in vivo metabolic networks. We performed KEGG pathway enrichment analysis to identify the major biochemical metabolic and signal transduction pathways involved in the differential metabolites. The top 20 enriched metabolic pathways after QCHS treatment are shown in Fig. 4G, with significant pathways including Arachidonic acid metabolism (P = 0.0009), Biosynthesis of unsaturated fatty acids (P = 0.0010), Linoleic acid metabolism (P = 0.0335), and Ferroptosis (P = 0.0398). Interestingly, all these pathways are closely related to lipid metabolism and ferroptosis. Next, we analyzed the relative abundance of differential metabolites in these four metabolic pathways and found that QCHS significantly increased the level of Glutathione (GSH), which is a key regulator of ferroptosis (Fig. 4H). This metabolomic analysis suggests that QCHS may influence GSH-mediated ferroptosis metabolism in colitic mice.

### *L. gasseri* is significantly correlated with ferroptosis metabolism

**Fig. 5:**
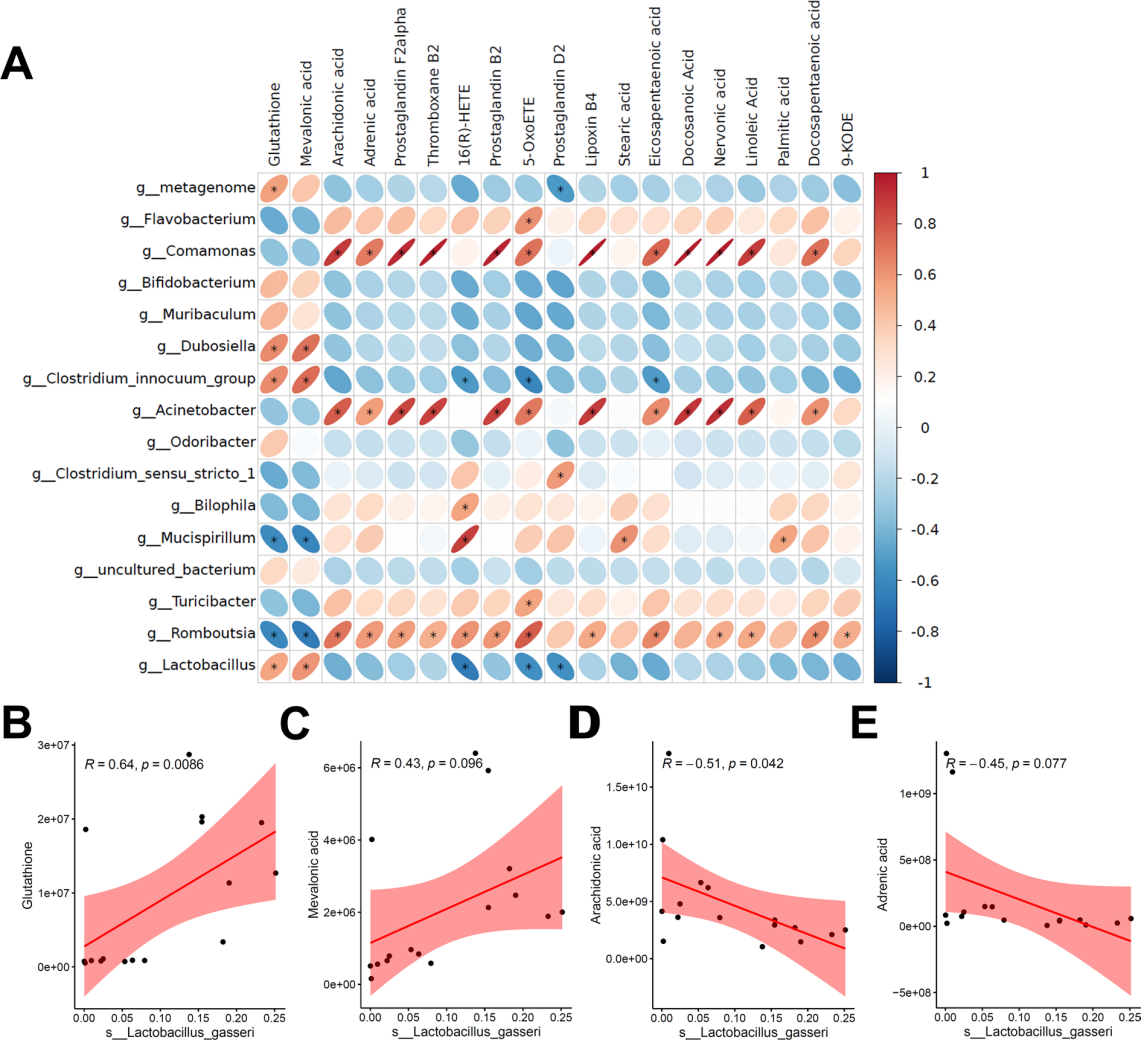
Correlation analysis of *L. gasseri* and ferroptosis metabolism. **A** Correlation analysis of significantly different metabolites and significantly different bacteria in feces. **B-E** Correlation analysis between metabolite levels in ferroptosis metabolic pathway and paired *L. gasseri* abundance in feces. All data are shown as mean ± SEM. *P < 0.05 (spearman analysis)

To explore the association between gut microbiota and metabolites affected by QCHS, we performed Pearson correlation analysis on the metabolites and differential bacteria at the genus level. The results showed that *Lactobacillus* was positively correlated with metabolites of the ferroptosis pathway (glutathione and mevalonic acid) and negatively correlated with metabolites in arachidonic acid metabolism (5-oxoicosatetraenoic acid, prostaglandin D2, and 16(R)-hydroxyeicosatetraenoic acid) (Fig. 5A). Next, Spearman-based correlation analysis was performed between QCHS-mediated *L. gasseri* and four metabolites of the ferroptosis metabolic pathway. The results indicated that the relative abundance of *L. gasseri* was significantly positively correlated with GSH and negatively correlated with mevalonic acid (MVA) (Fig. 5B–E), suggesting that QCHS-mediated *L. gasseri* may potentially inhibit ferroptosis.

### QCHS inhibited DSS-induced ferroptosis damage in UC mice

**Fig. 6:**
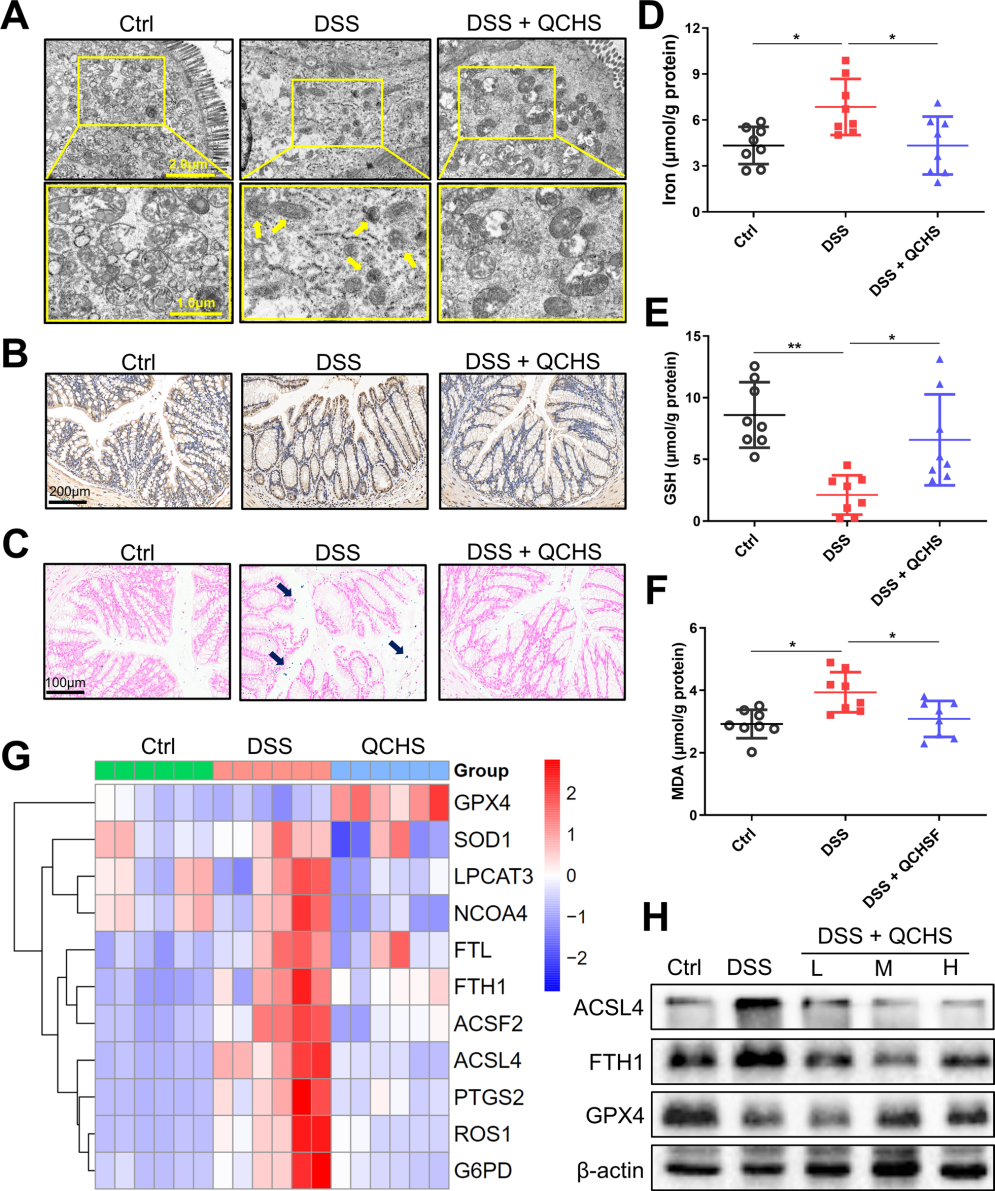
QCHS attenuated DSS-induced ferroptosis in mice. **A** Mitochondrial structure in colonic epithelial cells from UC mice were determined using transmission electron microscopy with yellow arrows indicating shrunken and disrupted mitochondria, low‑magnification view, scale bar: 2.0 μm; high‑magnification view, scale bar: 1.0 μm. **B** Immunohistochemical staining was used to detect the expression of 4-HNE, Scale bar: 200 μm. **C** The intracellular iron deposition was detected using Prussian blue staining, Scale bar: 100 μm. **D** Iron assay kit was used to detect the levels of intracellular iron in the colon. **E** GSH levels of the colon tissue were measured by GSH assay. **F** MDA levels of the colon tissue were measured by MDA assay. **G** The mRNA levels of ferroptosis-related genes were analyzed by real-time PCR. **H** The levels of ferroptosis-related proteins (ACSL4, FTH1, and GPX4) were examined by immunoblotting. All data are shown as mean ± SEM. *P < 0.05, **P < 0.01, ***P < 0.001(one-way ANOVA)

The results of the metabolomic analysis show that QCHS significantly enriched ferroptosis metabolism in the feces. Based on these findings, we hypothesized that the therapeutic effect of QCHS in UC mice might be mediated through ferroptosis. To verify this hypothesis, we collected colon tissue and serum from UC mice for various assays. Electron microscopy analysis revealed shrunken mitochondria and reduced mitochondrial cristae in colonic epithelial cells after DSS induction, whereas QCHS significantly alleviated this ferroptosis-like injury in the mitochondria (Fig. 6A). Lipid peroxidation plays a key role in the process of ferroptosis. 4-Hydroxynonenal (4-HNE), the end product of lipid peroxidation, is used as an indicator to detect lipid peroxidation. Immunohistochemical results showed that QCHS significantly reduced the abnormal increase of 4-HNE induced by DSS (Fig. 6B). Additionally, lipid peroxidation, as indicated by MDA levels, was elevated after DSS induction but significantly decreased following QCHS treatment (Fig. 6F). Ferroptosis is also dependent on the accumulation of iron ions. Both Prussian blue staining and iron assays demonstrated that QCHS reduced the iron overload caused by DSS (Fig. 6C–D). We also assessed the mRNA levels of ferroptosis-related genes and found that their expression was upregulated following DSS induction, but remarkably decreased after QCHS treatment. Conversely, the levels of GSH and the catalytic enzyme GPX4 were significantly reduced in the colon of UC mice. Interestingly, QCHS promoted the expression of both GSH and GPX4 (Fig. 6E and G). Moreover, after DSS induction, the protein levels of ACSL4 and FTH1, which are characteristic of ferroptosis, were significantly elevated, while GPX4 levels were reduced. QCHS treatment effectively reversed the levels of these ferroptosis-related proteins (Fig. 6H). This data suggests that QCHS exerts a protective effect against DSS-induced ferroptosis in the colon.

### QCHS promoted the proliferation of *L. gasseri* in vitro

**Fig. 7:**
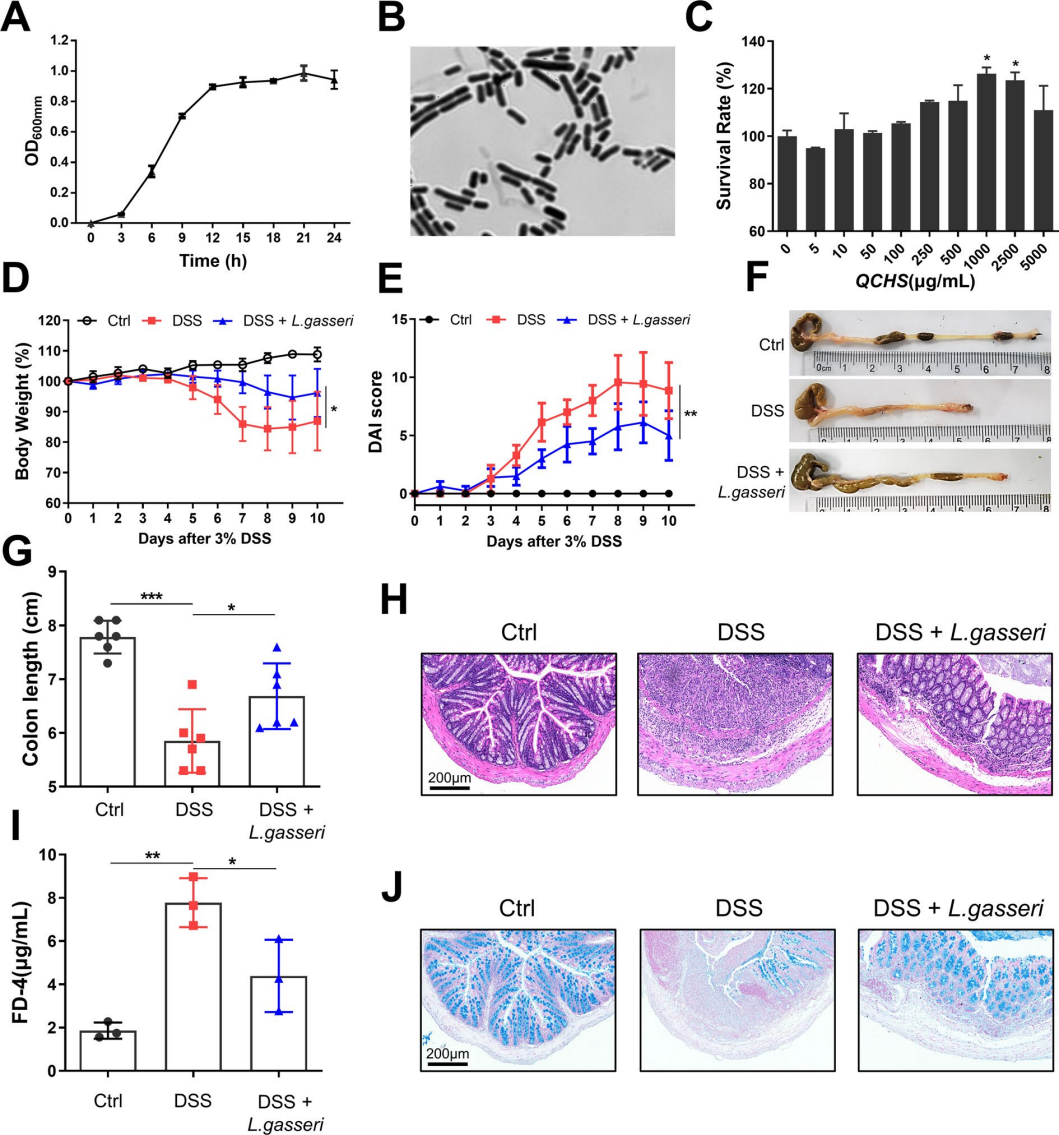
QCHS-mediated L. gasseri alleviated DSS-induced intestinal injury in mice. **A** Growth curves of *L. gasseri* at 37 °C for 24 h in MRS broth. **B** Gram-stained *L. gasseri* viewed under 63 × magnification. **C** The effect of QCHS on *L. gasseri* survival was analyzed. DSS-induced colitis mouse model was used to evaluate the effect of *L. gasseri.* **D** Body weight change, **E** Disease activity index (DAI) and **F-G** Colon length were evaluated. **H** H&E staining and pathological scoring of the colon, Scale bar: 200 μm. **I** Intestinal permeability was measured by FITC-Dextran 4 (FD-4) concentration in the serum. **J** Alcian blue staining of colon tissue, scale bar: 200 μm. All data are shown as mean ± SEM. *P < 0.05, **P < 0.01, ***P < 0.001 (one-way ANOVA)

16S rDNA sequencing results showed that QCHS increased the relative abundance of *L. gasseri* in the feces of UC mice, and the level of *L. gasseri* exhibited a significant positive correlation with GSH in the ferroptosis pathway. To further investigate the relationship between QCHS and *L. gasseri*, we cultured the *L. gasseri* strain with MRS medium in vitro (Fig. 7A–B). Notably, the combination of QCHS (1000 μg/mL) and MRS medium significantly promoted the growth of *L. gasseri* (Fig. 7C).

### *L. gasseri *reduced DSS-induced intestinal injury in mice

Based on the above results, we speculated that the protective effect of QCHS on UC mice might be mediated by *L. gasseri*. To test this hypothesis, we administered *L. gasseri* (5 × 10^8^ CFU) to mice with or without DSS induction for 7 days to determine whether *L. gasseri* could influence the progression of UC in mice. *L. gasseri*-treated mice exhibited reduced body weight loss, lower DAI scores, restored colon length, and alleviated pathological damage compared to mice treated with DSS alone (Fig. 7D–H). While DSS-treated mice showed higher serum FD-4 levels and reduced colonic mucin secretion, *L. gasseri* treatment significantly mitigated DSS-induced colonic barrier damage (Fig. 7I–J). These findings suggest that *L. gasseri* can slow the progression of colitis and protect against intestinal injury in UC mice.

### *L. gasseri* inhibited DSS-induced ferroptosis-like injury in mice

**Fig. 8:**
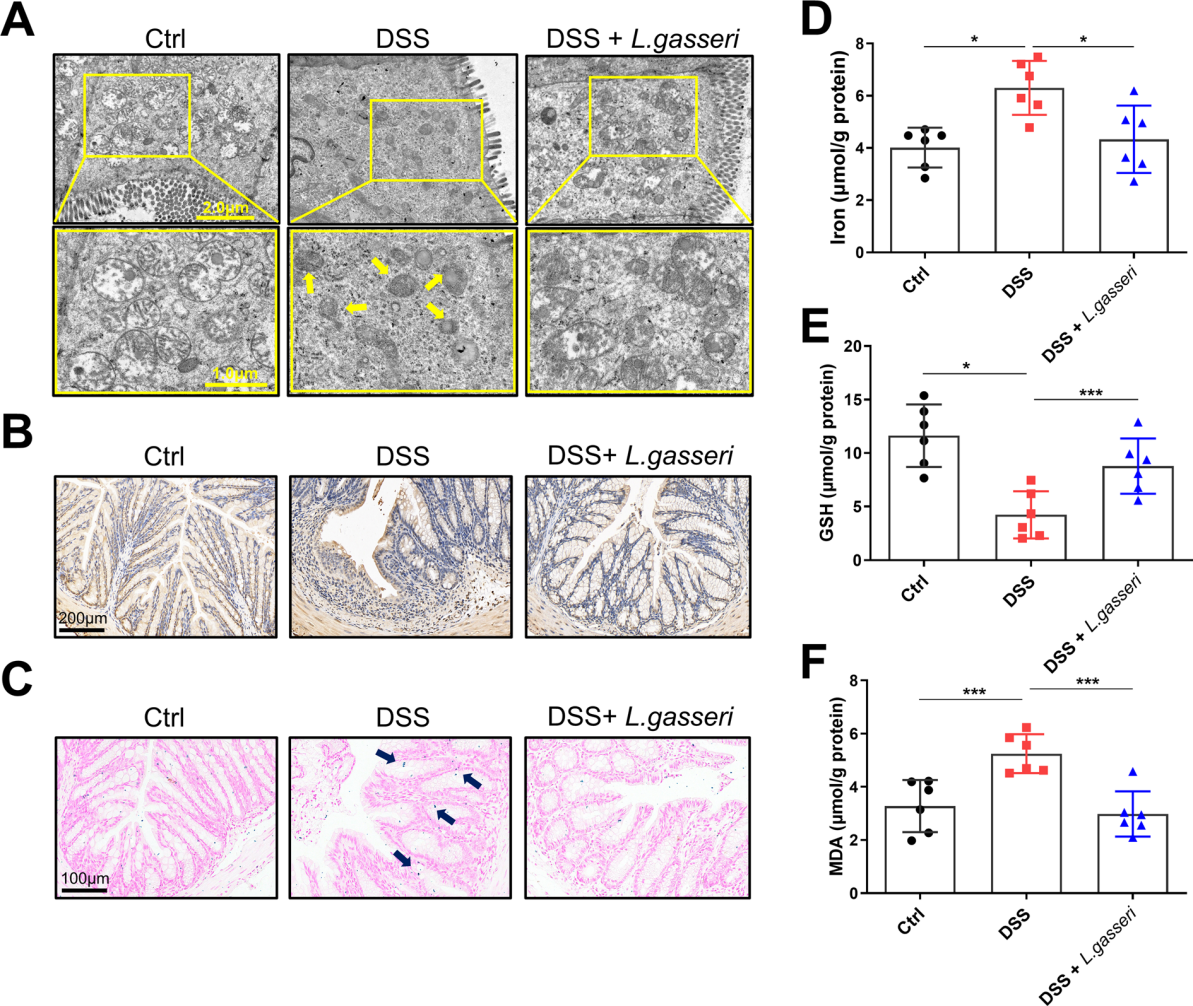
*L. gasseri* reduced DSS-induced ferroptosis in mice. **A** Mitochondrial structure in colonic epithelial cells from UC mice were determined using transmission electron microscopy with yellow arrows indicating shrunken and disrupted mitochondria, Scale bar: 1.0–2.0 μm. **B** Immunohistochemical staining was used to detect the expression of 4-HNE, Scale bar: 200 μm. **C** The intracellular iron deposition was detected using Prussian blue staining, Scale bar: 100 μm. **D** Iron assay kit was used to detect the levels of intracellular iron in the colon. **E** GSH levels of the colon tissue were measured by GSH assay. **F** MDA levels of the colon tissue were measured by the MDA assay. All data are shown as mean ± SEM. *P < 0.05, **P < 0.01, ***P < 0.001 (one-way ANOVA)

We also examined colonic ferroptosis impairment in *L. gasseri*-treated UC mice. Electron microscopy images showed that shrunken mitochondria and reduced mitochondrial crista in colonic epithelial cells could be significantly alleviated by treatment with *L. gasseri* (Fig. 8A). 4-HNE staining and Prussian blue staining of colon tissue showed that *L. gasseri* alleviated lipid peroxidation and excessive accumulation of iron ions caused by DSS, respectively (Fig. 8B–C). In addition, the *L. gasseri* treatment increased the levels of GSH, while effectively decreasing the levels of iron and MDA in DSS-induced colitis (Fig. 8D–F). Therefore, *L. gasseri* may attenuate intestinal injury in colitic mice by inhibiting ferroptosis.

### *L. gasseri* inhibited LPS-induced inflammation in NCM-460 cells

**Fig. 9:**
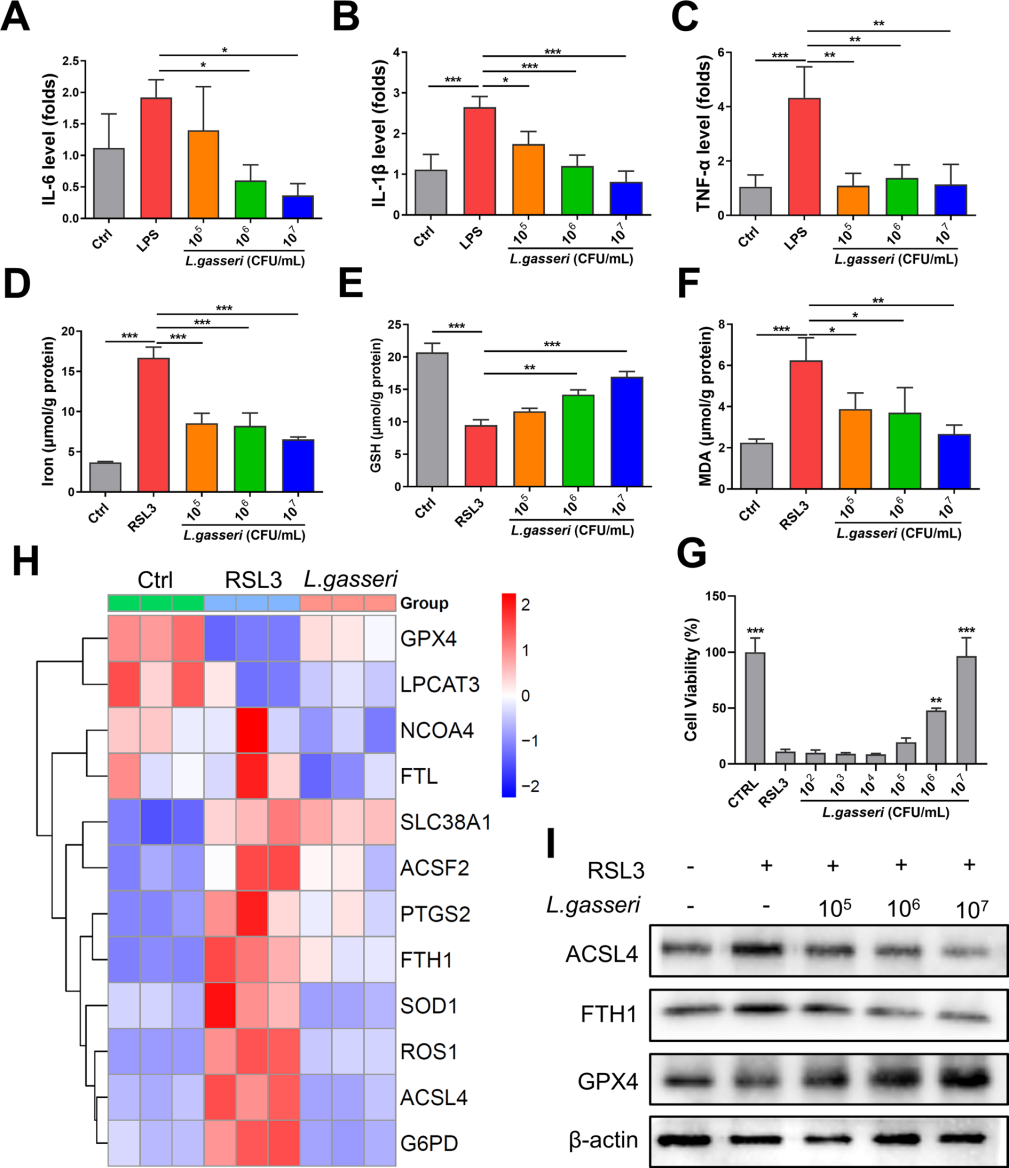
*L. gasseri* inhibited inflammation and RSL3-induced ferroptosis in NCM-460 cells. **A**–**C** NCM-460 cells were pretreated with *L. gasseri* for 12 h and then stimulated with or without LPS (500 ng/mL). The mRNA levels of IL-6, IL-1β, and TNF-α were detected by real-time PCR. **D**–**F** RSL3 was utilized to induce ferroptosis in NCM460 cells after *L. gasseri* treatment and the level of intracellular iron, GSH, and MDA of NCM-460 cells were detected. **G** Cell viability was analyzed by the CCK-8 assay. **H** Total RNA was extracted and the mRNA levels of ferroptosis-related genes were analyzed by real-time PCR. **I** The protein levels of ACSL4, FTH1, and GPX4 were examined by western blotting. All data are shown as mean ± SEM. *P < 0.05, **P < 0.01, ***P < 0.001 (one-way ANOVA)

Next, we investigated the effect of *L. gasseri* on inflammation and ferroptosis through in vitro experiments. First, NCM-460 cells were pretreated with the indicated concentration of *L. gasseri* for 12 h, followed by the addition of LPS (1 μg/mL) to induce cellular inflammation. As expected, *L. gasseri* significantly reduced the mRNA levels of IL-6, IL-1β, and TNF-α in LPS-induced NCM-460 cells (Fig. 9A–C).

### *L. gasseri* inhibited RSL3-induced ferroptosis in NCM-460 cells

Finally, RSL3, a known inhibitor of GPX4, was used to induce ferroptosis in NCM-460 cells. As shown in Fig. 9D–F, RSL3 significantly increased the levels of iron and MDA, while decreasing GSH content in NCM-460 cells. However, pretreatment with *L. gasseri* effectively prevented iron overload and lipid peroxidation, and significantly increased GSH levels in the cells (Fig. 9D–F). More importantly, the cell viability assay revealed that *L. gasseri* pretreatment significantly inhibited RSL3-induced cell death (Fig. 9G). Additionally, analysis of ferroptosis-related molecules showed that *L. gasseri* increased the expression of GPX4 at both the mRNA and protein levels, while significantly inhibiting the positive ferroptosis regulators such as FTH1 and ACSL4 (Fig. 9H–I). These results suggest that *L. gasseri* may act as a potential ferroptosis inhibitor, offering protection against cellular injury.

## Discussion

The incidence of ulcerative colitis (UC) has been rising steadily in developing countries, placing a considerable burden on healthcare systems [27, 28]. Given the challenges of controlling inflammation and promoting mucosal repair in UC treatment, there is a pressing need for the development of new therapeutic strategies. Traditional Chinese medicine (TCM) prescriptions, including QCHS, are increasingly being recognized as multi-targeted therapies. Key components identified in QCHS include berberine, baicalin, coumarin, ferulic acid, and paeoniflorin, among others. Detailed information about these chemical components has been provided in our previous study [25]. A randomized clinical trial has confirmed the efficacy and safety of QCHS in patients with moderately active UC [23]. However, the specific mechanisms through which QCHS exerts its effects remain largely unexplored.

Dysbiosis in the gut microbiota is known to disrupt immune homeostasis, leading to abnormal immune responses and inflammatory cytokine release [29, 30]. Inflammatory bowel disease (IBD) patients exhibit altered gut microbial diversity, such as an imbalance between *Firmicutes* and *Bacteroidetes*, an increase in *Proteobacteria*, and the depletion of *Roseburia* species [31, 32]. The mucus layer, which interacts with both the microbiota and immune cells, plays a critical role in maintaining gut homeostasis. However, the expansion of pathogenic bacteria can break down this barrier, leading to “leaky gut” and increasing the risk of pathogens entering the lamina propria and bloodstream [33–35]. Dysbiosis may also disrupt the metabolome, further compromising the mucosal barrier and contributing to inflammation. For instance, the depletion of short-chain fatty acids (SCFAs) promotes the polarization of M1 macrophages, which drives intestinal inflammation [36]. Additionally, fecal metabolism of palmitoleic acid and tryptophan degradation have been linked to the production of TNF-α and interferon-gamma (IFN-γ), both of which are associated with inflammation [37].

Probiotics have been shown to restore microbial diversity, and their therapeutic potential in IBD has been demonstrated in both clinical and animal studies [38, 39]. *Lactobacillus* species, in particular, are among the most widely used probiotics, and their depletion is associated with IBD progression [40, 41]. *Lactobacillus* helps repair the intestinal epithelial barrier by upregulating tight junction proteins and reduces colonic inflammation in UC mice by inhibiting the TLR4-NF-κB-NLRP3 signaling pathway [42, 43]. Notably, prebiotic therapy has been shown to promote probiotic growth, restoring gut function and alleviating IBD symptoms [44, 45].

In this study, we demonstrated that QCHS treatment alleviated DSS-induced colitis by reducing colitis symptoms, suppressing pro-inflammatory cytokine secretion, and repairing the colonic epithelial barrier. These findings are consistent with previous reports showing that traditional herbal formulations can attenuate mucosal inflammation and restore epithelial integrity in UC models [46, 47]. To further elucidate the underlying mechanisms of QCHS, we employed microbial sequencing and metabolomic analysis. Our data revealed that QCHS reshaped the gut microbiota, with a notable increase in the relative abundance of *Lactobacillus gasseri* in the feces of UC mice. This observation is particularly significant, as earlier studies have highlighted the protective role of *Lactobacillus* species in maintaining intestinal homeostasis and reducing colitis severity [48, 49], but none have directly linked *L. gasseri* to ferroptosis regulation. In vitro experiments confirmed that QCHS directly promoted the growth of *L. gasseri* strains, supporting a causal link between QCHS administration and microbial enrichment.

Untargeted metabolomics demonstrated that QCHS altered the fecal metabolic profile, with differential metabolites significantly enriched in ferroptosis-related pathways. Correlation analysis and in vitro experiments further demonstrated that *L. gasseri* suppressed RSL3-induced ferroptosis via activation of the GSH/GPX4 pathway.

The GPX4/GSH axis is a central regulator of ferroptosis, a form of regulated cell death (RCD) that is closely associated with lipid peroxidation. Disruptions in iron metabolism leads to the accumulation of intracellular free iron, which catalyzes the generation of reactive oxygen species (ROS) through the Fenton reaction. ROS then promote lipid peroxidation and trigger ferroptosis [50]. Additionally, polyunsaturated fatty acids (PUFAs), which are substrates for lipid peroxidation, influence ferroptosis susceptibility [51]. Our data showed that QCHS enriched pathways involved in arachidonic acid metabolism, biosynthesis of unsaturated fatty acids, and linoleic acid metabolism. Specifically, QCHS reduced the abundance of arachidonic acid, adrenic acid, linoleic acid, 16(R)-HETE, palmitic acid, prostaglandin F2α, prostaglandin D2, and other metabolites, which are implicated in both lipid peroxidation and inflammation. For example, arachidonic acid induces inflammation through its conversion to prostaglandins (PGs) via the cyclooxygenase (COX) pathway [52].

Emerging evidence suggests that ferroptosis plays a critical role in the pathogenesis of IBD, and inhibiting ferroptosis may offer a novel therapeutic approach for UC [53, 54]. Our study demonstrated that QCHS significantly alleviated DSS-induced ferroptosis in the colon of UC mice, and represents the first evidence linking *L. gasseri* to ferroptosis regulation. In vitro, we demonstrated that *L. gasseri* inhibited RSL3-induced ferroptosis in NCM-460 cells, with the mechanism involving activation of the GSH/GPX4 signaling pathway. While ferroptosis has been implicated in UC pathogenesis, the involvement of commensal bacteria in modulating this pathway has not been reported. We hypothesize that *L. gasseri* may act as a ferroptosis inhibitor by producing GSH precursors or stimulating host cell GSH synthesis pathways (e.g., via the Nrf2 signaling pathway [55]), thereby enhancing GSH synthesis or GPX4 activity and protecting epithelial cells from lipid peroxidation. While our data strongly suggest that *L. gasseri* enrichment is a key mechanism, future studies using germ-free models or specific *Lactobacillus*-depletion strategies are needed to confirm its indispensability for QCHS’s full therapeutic effect.

**Fig. 10:**
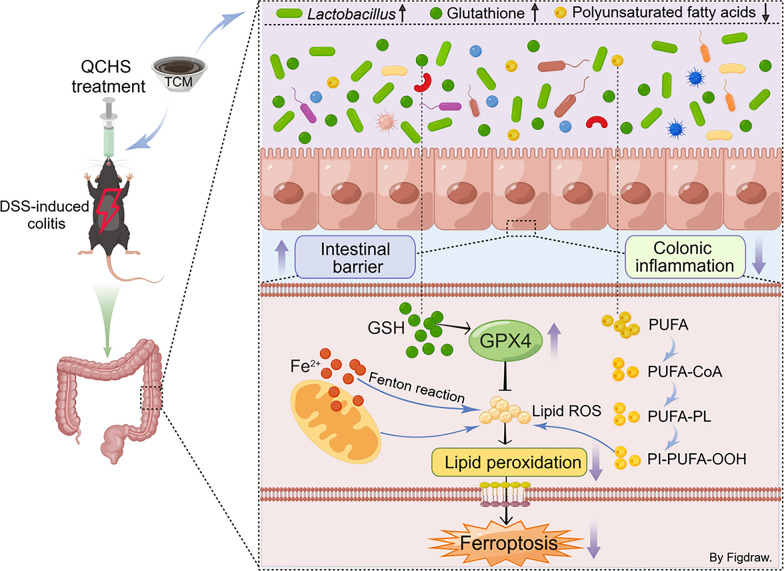
Graphic abstract for the mechanism of QCHS

Taken together, our study provides compelling evidence for the regulatory role of QCHS on the microbiota-metabolome axis and ferroptosis in UC mice (Fig. 10). We also uncover a novel function of *L. gasseri* as an inhibitor of ferroptosis, offering new insights into potential therapeutic strategies for UC. These findings suggest that through the microbiota modulators or ferroptosis inhibitors targeting *Lactobacillus*, QCHS may be promising candidates for the treatment of UC.

## Data Availability

No datasets were generated or analysed during the current study.

## Abbreviations

UC: Ulcerative colitis
QCHS: Qing-Chang-Hua-Shi granule
*L. gasseri*: *Lactobacillus gasseri*
IBD: Inflammatory bowel disease
SCFAs: Short-chain fatty acids
IL: Interleukin
RCD: Regulated cell death
ROS: Reactive oxygen species
GPX4: Glutathione peroxidase 4
GSH: Glutathione
System Xc−: The cystine/glutamate antiporter system
TCM: Traditional Chinese medicine
DSS: Dextran sulfate sodium
DAI: Disease activity index
TNF-α: Tumor necrosis factor-α
Lcn2: Lipocalin 2
FD-4: FITC-Dextran 4
Muc2: Mucin2
4-HNE: 4-Hydroxynonenal
MDA: Malondialdehyde
ACSL4: Acyl-CoA synthetase long-chain family member 4
FTH1: Ferritin heavy polypeptide 1
GSH: Glutathione
KEGG: Kyoto Encyclopedia of Genes and Genomes
MVA: Mevalonic acid
IFN-γ: Interferon gamma
PUFAs: Polyunsaturated fatty acids
PGs: Prostaglandins
